# Meta-analysis to estimate the load of Leptospira excreted in urine: beyond rats as important sources of transmission in low-income rural communities

**DOI:** 10.1186/s13104-017-2384-4

**Published:** 2017-01-28

**Authors:** Veronica Barragan, Nathan Nieto, Paul Keim, Talima Pearson

**Affiliations:** 1 Pathogen & Microbiome Institute, Northern Arizona University, Flagstaff, AZ 86011-4073 USA; 2 Department of Biological Sciences, Northern Arizona University, Flagstaff, AZ 86011-5640 USA; 3 Instituto de Microbiologia, Colegio de Ciencias Biologicas y Ambientales, Universidad San Francisco de Quito, Quito, Ecuador

**Keywords:** Leptospira, Animal reservoirs, Urine, Transmission

## Abstract

**Background:**

Leptospirosis is a major zoonotic disease with widespread distribution and a large impact on human health. Carrier animals excrete pathogenic Leptospira primarily in their urine. Infection occurs when the pathogen enters a host through mucosa or small skin abrasions. Humans and other animals are exposed to the pathogen by direct contact with urine, contaminated soil or water. While many factors influence environmental cycling and the transmission of *Leptospira* to humans, the load of pathogenic *Leptospira* in the environment is likely to play a major role. Peridomestic rats are often implicated as a potential source of human disease; however exposure to other animals is a risk factor as well. The aim of this report is to highlight the importance of various carrier animals in terms of the quantity of *Leptospira* shed into the environment. For this, we performed a systematic literature review and a meta-analysis of the amount of pathogen that various animal species shed in their urine.

**Results:**

The quantity of pathogen has been reported for cows, deer, dogs, humans, mice, and rats, in a total of 14 research articles. We estimated the average *Leptospira* per unit volume shed by each animal species, and the daily environmental contribution by considering the total volume of urine excreted by each carrier animal. Rats excrete the highest quantity of *Leptospira* per millilitre of urine (median = 5.7 × 10^6^ cells), but large mammals excrete much more urine and thus shed significantly more *Leptospira* per day (5.1 × 10^8^ to 1.3 × 10^9^ cells).

**Conclusions:**

Here we illustrate how, in a low-income rural Ecuadorian community, host population demographics, and prevalence of *Leptospira* infection can be integrated with estimates of shed *Leptospira* to suggest that peridomestic cattle may be more important than rats in environmental cycling and ultimately, transmission to humans.

**Electronic supplementary material:**

The online version of this article (doi:10.1186/s13104-017-2384-4) contains supplementary material, which is available to authorized users.

## Background

Leptospirosis is a zoonotic disease caused by spirochete bacteria in the genus *Leptospira*. Early stages of human leptospirosis are characterized by non-specific symptoms such as headaches, high fever, jaundice, and mucosal hemorrhages; severe disease may produce multisystem complications such as acute renal or hepatic failure, or severe pulmonary hemorrhaging among other pathologies [1]. A variety of animals including rats, horses, cattle, dogs, pigs [2–5], and numerous wild life species such as bats, coyotes, raccoons, sea lions, opossums, coyotes, white-tailed deer and even frogs and caimans [6–11] have also been shown to carry pathogenic *Leptospira*. Upon infection, *Leptospira* bacteria become particularly concentrated in the kidneys and genital tracts [12] where they can be shed into the environment via urine. As such, any infected human or animal can potentially infect others directly or indirectly by contaminating the environment. Outside a host, pathogenic *Leptospira* can survive in soil and water [13, 14]. Transmission can occur when contaminated urine, soil, or water comes into contact with exposed mucosa, wounded skin or when ingested [14, 15].

Human and animal leptospirosis outbreaks are most commonly reported in tropical rural and urban slums [1, 16–18], however they also occur in cities throughout the world [18–21]. In urban areas, where most studies have been conducted [15], rats and dogs are common and have often been identified as potential sources of human infection [1, 2, 5, 14, 22–25]. Contact with other animals, such as livestock, is commonly regarded as an occupational, rather than peridomestic risk factor [26, 27]. However, in rural areas, contact with a variety of animals and livestock can be more common and therefore not restricted to occupational exposure [28, 29]. In many agrarian and pastoralist communities, families live in close proximity to their animals, increasing the likelihood of peridomestic contact for all family members. In tropical developing countries, up to 65% of humans live in rural areas [30], and despite the likely importance of a diverse array of potential animal hosts and the impact of the environment, the role of rats is perhaps overrepresented in the peer-reviewed and public health literature.

Our aim here is to provide a focused meta-analysis to explore the potential importance of a variety of animals in shedding *Leptospira* into the environment. In doing this, we focus on species-specific estimates of the amount of *Leptospira* shed in urine. To illustrate the potential load of Leptospira shed into the environment via cattle urine, we combined prevalence and demographic data from a highly endemic rural community in Ecuador with quantitative shedding estimates from individual animals. We thus discuss the importance of host densities in determining the overall quantity of *Leptospira* shed into the environment. Given the paucity of data on many animals, our analysis is restricted to a small number of peridomestic species and one wild species. The role of different animals in the environmental cycling of these pathogens is likely regionally and culturally specific and may be impacted by the dynamic nature of *Leptospira* strain or species prevalence. However, knowledge of the potential roles of a variety of animals is essential for estimating risks posed by different host species towards a better understanding of conditions under which disease or outbreaks are most likely.

## Methods

### Quantifying shed *Leptospira*

We searched Pubmed (http://www.ncbi.nlm.nih.gov/pubmed) and Web of Science (http://apps.webofknowledge.com) on October 24th, 2015 using the terms “*Leptospira* AND ((Shedding) OR (Excretion) OR (Leptospiruria))” without restrictions on publication date. We retrieved 110 titles from Web of Science and 125 from Pubmed. Removing duplicates left 156 total. By screening abstracts, we excluded 126 papers that were not about leptospirosis, did not quantify *Leptospira* in urine, or were in languages other than English or Spanish. We further screened the 30 remaining papers to include only 14 that reported the quantity of *Leptospira* in urine of animals infected naturally or experimentally (Additional file 1: Figure S1). Quantity of shed *Leptospira* per millilitre by animal type was either extracted from manuscript figures using WebPlot Digitizer [31] or from manuscript tables. Quantity shed by dogs was kindly provided by Jarlath Nally and Pablo Rojas [32]. For each manuscript, we recorded characteristics of the quantification method: target gene, lowest limit of detection (lLoD), and *Leptospira* clade [17] specificity of assays (Table 1). *Leptospira* load per millilitre of urine was registered for each animal type (Additional file 2: Table S1).

**Table 1:** Techniques used to measure quantity of *Leptospira* in urine

Target Gene	Leptospira clade^a^	Method	lLoD^b^	Reference
–	All Leptospira species	Darkfield microscopy	Semiquantitative	Nally et al. [45]; Monahan et al. [46]
*Non identified*	Pathogenic clade	Conventional PCR	Semiquantitative	Gerritsen et al. [47, 48]
**–**	Pathogenic clade	Slot blot -Scanning laser densitometry	Semiquantitative	Zuerner et al. [49]
*16S rrna*	Pathogenic clade Intermediate clade	TaqMan PCR	10 cells/mL of urine	Smyth et al. [50, 51]
*lipl32*	Pathogenic clade	TaqMan PCR	10^1^ to 10^2^ cells/mL of urine	Sttodard et al. [52]
*lipl32*	Pathogenic clade	TaqMan PCR	3GEq/4.5 μL of extracted DNA	Rojas et al. [32]
*lipl32*	Pathogenic clade	TaqMan PCR	6 Geq/5 μL of extracted DNA	Villumsen et al. [53]
*gyrB*	Pathogenic clade	SYTO9 PCR	10^3^ cells/mL	Subharat et al. [54]

^a^ *Leptospira* species or clade as designated according to Levett [17]

^b^Lowest limit of detection as reported by authors

### Host comparisons

We performed a Kruskal–Wallis test [33] to assess differences in the quantity of *Leptospira* shed among animal species (cattle, deer, dogs, humans, rats, and mice). To test whether the quantification method (qPCR—quantitative PCR, scanning laser densitometry, gel quantification, or dark field microscopy enumerations) caused differences in the mean *Leptospira* quantity, we compared results from within a host species across quantification methods using the Wilcoxon Rank Sum Test [34]. *Leptospira* load per millilitre of urine were transformed to Log base 10 for data analysis. Average volume of urine shed per animal was calculated from the literature: cattle [35], deer [36], dogs [37], humans [38], mice [39], and rats [40] (Additional file 3: Table S2).

### Estimation of *Leptospira* quantity shed by cattle in an endemic rural community

In a previous study [41] we found that 35.4% of cows living in Abdon Calderon Parish in Manabi province (Ecuador) were shedding Leptospira DNA in their urine. The Ecuadorian Ministry of Agriculture conducted the most recent census in 2000 (http://sinagap.agricultura.gob.ec/censo-nacional-agropecuario). Data from this census, contained in the Ministerio de Agricultura, Ganaderia, Acuacultura y Pesca (MAGAP) database [42], showed a total of 78 properties in Abdon Calderon with a total of 886 cattle. We calculated the contribution of *Leptospira* from cattle by modifying the formula used by Costa et al. [5]: DPC = PS*Prev*Vol*Load, where DPC = Daily population contribution, PS = Population size (number of cattle per property size; 0.35–1, 1–5, 5–10 ha, and more than 10 ha), Prev = prevalence in the given population (35.4%), Vol = is the average volume of urine shed per day, and load is defined as log of cell/mL. Given that the average volume of urine shed per urination event is 2 L [35] and the average number of urination events per day is 7–12 [43, 44], we calculated Vol = 16 L.

## Results

### Quantity of *Leptospira* shed in urine

We identified fourteen articles that quantified pathogenic *Leptospira* in urine from experimentally or naturally infected animals. Quantification methods included dark-field microcopy, scanning laser densitometry, gel electrophoresis, and qPCR (Additional file 2: Table S1). Five different qPCR assays have been used to quantify *Leptospira* in urine of experimental or naturally infected animals (Table 1). Four of five qPCR assays target only species that belong in the pathogenic clade while one assay also detected infectious *Leptospira* from the “intermediate” clade. We found no significant differences in quantification methods among studies of cattle (W = 16, p = 0.095) and rats (W = 107, p = 0.238).

**Fig. 1:**
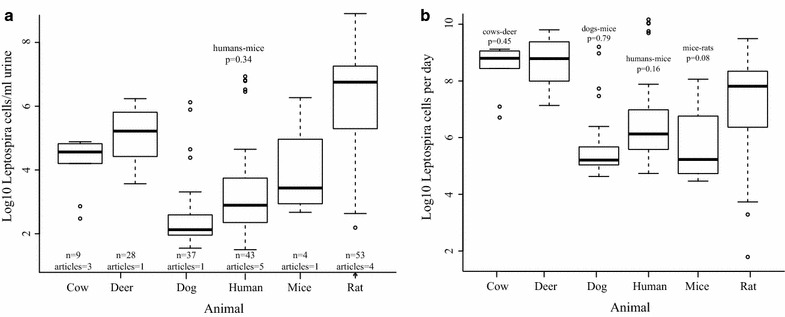
Quantity of Leptospira shed by animals. **a** Quantity of shed Leptospira per milliliter (Log10) of urine is significantly different among animals (Kruskal–Wallis Chi squared = 96.33, *p* value <2.2 × 10–16). Comparisons of quantity of Leptospira shed between pairs of animals were all significantly different except humans and mice (Kruskal–Wallis Chi squared = 0.91, p = 0.34). **b** Estimates of absolute quantity of Leptospira shed per day differ significantly among animals (Kruskal–Wallis Chi squared = 73.6, p = 1.806 × 10–14). Quantity of Leptospira shed per day by cattle and deer are significantly higher than dogs, humans, mice and rats (Kruskal–Wallis Chi squared = 45.6, p = 1.45 × 10–11). No significant differences were found when comparing cattle and deer, dogs and mice, humans and mice, and rats and mice. *Box-plots* display the medians, interquartile range (IQR), 1.5 × IQR, and suspected outliers >1.5 × IQR

Shed *Leptospira* have been quantified for cattle, deer, dogs, humans, mice and rats (Table 1; Additional file 2: Table S1). The quantity of pathogenic *Leptospira* shed per millilitre of animal urine differs significantly by species (Fig. 1a; Additional file 4: Table S3). The lowest quantity of *Leptospira* shed per millilitre of urine was calculated for humans (32 cells/mL) while the highest quantity was calculated for rats (8 × 10^8^ cells/mL). When estimating median absolute quantity of *Leptospira* shed per day, mice shed the least (1.9 × 10^5^ cells), and cattle and deer the highest with 6.3 × 10^8^ and 6.1 × 10^8^ cells, respectively (Additional file 4: Table S3).

### Daily population contribution of pathogenic *Leptospira* via cattle urine in an endemic rural community

The estimated daily quantity of pathogenic *Leptospira* shed by cattle in Abdon Calderon (Ecuador) was calculated using the local prevalence in cattle of 35.4% [41], demographic data collected by MAGAP [42], and daily quantity (median) of *Leptospira* shed by cattle (Additional file 4: Table S3). Grazing range characteristics are likely to play a role in the concentration of environmental *Leptospira* as well as the likelihood of direct or indirect contact of contaminated urine by humans. Some properties in this area are fenced and others are not, but such information was not registered in the census database, limiting our ability to make inferences about interactions of animals across properties or with wildlife. In Abdon Calderon, the lowest estimate (4.9 × 10^3^ cells/m^2^/day) of the amount of pathogenic *Leptospira* shed via cattle urine was associated with the lowest estimated density of cattle (1 animal in 4.2 ha). Conversely, given that some herds with as many as 40 cattle were confined to a grazing area of only 2 ha, we estimated the amount of pathogenic *Leptospira* shed via urine per day to be 4.2 × 10^5^ cells/m^2^/day. Importantly, 91 cattle at the study site live on properties without grazing areas (Table 2). These cattle are therefore moved through the community to drink and graze but are likely to spend much of their time confined to a very small area. These cattle may be shedding approximately 1.96 × 10^10^ cell/day, however we cannot estimate the area that they may contaminate.

**Table 2:** Daily population contribution of *Leptospira* (DPC) by cattle herds in Abdon Calderon, Manabi, Ecuador

Grazing area	Number of properties	Number of cattle	Quantity of *Leptospira* shed per m^2^
		Total (Min–Max)	Total (Min–Max)
No grazing area	18	91(1–20)	1.96 × 10^10^ cell/day
0.35 to 1 ha	9	26 (2–5)	4.2 × 10^4^ to 1.5 × 10^5^
>1 to 5 ha	30	219 (1–40)	4.9 × 10^3^ to 4.2 × 10^5^
>5 to 10 ha	10	176 (2–70)	5.9 × 10^3^ to 1.5 × 10^5^
More than 10 ha	10	354 (12–80)	9.2 × 10^3^ to 4 × 10^4^

## Discussion

A wide variety of animals can be infected with leptospira and might transmit the pathogen to humans, however the relative roles of each animal species is not well understood. Given the role of urine in seeding the environment with Leptospira, we illustrate how animal physiology and population data can be used to estimate the environmental load of the pathogen. Rats are traditionally thought to be the main reservoir for human transmission even though a variety of animals have also been implicated. Our results show that while rats may excrete the highest concentration of pathogen, the concentration, coupled with volume and animal density will dictate the total amount of pathogen in the environment. Our results illustrate how larger host species may play an important role in leptospirosis transmission and should not be overlooked.

Urine is the primary avenue for shedding *Leptospira* and thus plays a central role in the environmental cycling of this pathogen and infection risk [2]. Contact with contaminated urine, either directly or indirectly through contaminated soil or water can lead to transmission [14]. Many animals have been documented as competent hosts to *Leptospira*, but it is likely that these animals represent only a fraction of likely hosts that may play important roles in the environmental cycling and epidemiology of *Leptospira*. While contact with cattle and other livestock has been associated with transmission to humans, this interaction is mostly treated as an occupational risk, given that many studies were conducted in rural and urban slums where non-occupational animal contact mostly involves peridomestic rats and dogs. In many human populations, however, interactions within a diverse group of wildlife are common. Our aim here was to explore the potential roles of a variety of animals in *Leptospira* eco-epidemiology, illustrate how animal population data can be used to estimate the environmental load of *Leptospira*, and discuss other variables that may contribute to the likelihood of human infection.

We identified 14 research articles that quantified the amount of *Leptospira* shed in urine. These works were limited to six species and employed a number of different methods. As multiple articles employed different methods for quantifying *Leptospira* in urine from rats and cattle, we were able to determine that these different methods did not result in significant differences. Molecular methods may over-estimate quantity of *Leptospira* excreted in urine as they detect alive and dead bacteria, however microscopy quantification, detecting live cells shed by rats are within the range detected by qPCR, suggesting that the quantity of dead cells may not be significant. Furthermore, there is no evidence that this will affect relevant comparisons across species as performed in this meta-analysis. Among other variables, the absolute quantity of *Leptospira* shed per day by an infected animal depends on the quantity of pathogen in urine as well as the total daily volume of excreted urine. While rats may shed more *Leptospira* per unit volume of urine, the small overall volume of excreted urine limits their overall contribution to the environmental load. Larger animals such as cattle and deer shed less *Leptospira* per unit volume of urine, however the sheer volume of urine excreted by such animals can result in a significantly higher environmental contribution compared to rats and other animals. In some environments, however, extremely high rat densities will drastically increase the amount of urine shed into the environment. Therefore, in order to determine the overall contribution of an individual host species, population density and prevalence must also be considered.

There is little information on the prevalence of *Leptospira* in a given host species [55], and prevalence is likely to vary across regions and seasons [15, 56]. In 2014–2015, we estimated *Leptospira* prevalence among cattle (35.4%), pigs (5.7%), and rats (2.8%) in Abdon Calderon, Ecuador [41]. Demographic data on cattle ownership were not collected for this time period and the most recent data were collected in 2000. Undoubtedly population sizes have changed, however these data illustrate how demographic and prevalence data can be used to estimate the daily load of *Leptospira* shed per unit area. Given the availability of host population and leptospirosis prevalence data, models should ideally include multiple host species, including humans.

Animal behavior and animal husbandry practices will influence the load and distribution of pathogens shed into the environment as well as the likelihood of transmission to humans. Animal density will affect environmental load and our consideration of grazing area only provides a rough illustration of how shed *Leptospira* may be distributed. Cattle are gregarious, and even when provided a large grazing area, may spend a large portion of their time concentrated in small areas associated with bedding, feeding and watering, resulting in uneven distribution of shed *Leptospira*. Animal husbandry practices may increase the likelihood of human contact with shed *Leptospira*. Many cattle owners (23%) in Abdon Calderon do not own property on which to graze their herd. These animals (10.5% of the total cattle population) graze in public areas and are thus not segregated from the general human population. Also, these animals will spend significant amounts of time in the small peridomestic environment, increasing contact with family members, and presenting a non-occupational risk of infection. Similarly, humans may be more likely to come into contact with *Leptospira* shed from other humans. Human prevalence rates may be underestimated if only symptomatic patients are considered, and an infected human may shed 1.3 × 10^6^ cells per day. Human shedding may not play a significant role in the environmental cycling and transmission of *Leptospira* in places with good sewage infrastructure and available toilet facilities, however such infrastructure is lacking in most of the world. More complex modeling of *Leptospira* shedding must incorporate higher-resolution estimations of distributional variation and how shed *Leptospira* may come into contact with other animals and ultimately, humans.

Climatic variation is likely to result in temporal changes in leptospirosis prevalence among humans [13, 57] and other animals. Climate and weather can impact host population sizes, distribution, behaviors, and interactions. Environmental conditions can also affect survivorship and environment distribution of shed *Leptospira*. Indeed, *Leptospira* have been shown to survive best in soil with high relative humidity and neutral pH [58, 59]. Flooding and heavy rainfall have been associated with some leptospirosis outbreaks, but even during droughts, stagnant water or ponds may serve as refugia for *Leptospira* [60–62]. In Abdon Calderon across 2014–2015, recorded flooding events were rare and the local Health Ministry authorities reported isolated leptospirosis cases and no outbreaks. Flooding may serve as the main mechanism for distribution of shed *Leptospira*, providing a means for contacting *Leptospira* shed from animals that may not typically be transmitted between certain host species.

Lastly, the high genetic heterogeneity among *Leptospira* has resulted in variation in virulence and a certain degree of host adaptation [2]. It is also likely that certain species or genotypes may have differential environmental survivorship. Fourteen out of 21 *Leptospira* species cause disease, and within them, more than 200 serovars have been described [17]. Knowledge of circulating genotypes must certainly play a role in epidemiological modeling of *Leptospira*.

## Conclusion

We have focused this illustration on cattle; population, infection prevalence, and the quantity of *Leptospira* shed for many species are not available, and the high prevalence and high estimated daily shedding suggests that cattle in Abdon Calderon may have been the most important source of *Leptospira* in 2014–2015. However, more thorough modeling of environmental loads and the likelihood of direct/indirect human contact with urine must consider multiple host species, host behavior or animal husbandry practices that increase the likelihood of transmission to humans or other animals, and circulating pathogen genotypes that may differentially impact host species. To our knowledge, there are no reports that directly link an infected animal to a human leptospirosis case. Therefore, epidemiological investigations coupled with genotyping data of the pathogen will provide valuable insights into the roles of different animals in leptospirosis transmission and will confirm or refute our hypothesis of the importance of urine volume for *Leptospira* load in the environment and risk for human health.

## Additional files

**Additional file 1: Figure S1.** Study flow diagram.

**Additional file 2**: **Table S1.** Leptospira quantity data extracted from the articles analyzed in this meta-analysis.

**Additional file 3: Table S2.** Urine volume excreted by animals.

**Additional file 4: Table S3.** Estimated quantity of Leptospira shed by animals.

## Abbreviations

lLoD: lowest limit of detection
qPCR: quantitative PCR
MAGAP: Ministerio de Agricultura, Ganaderia, Acuacultura y Pesca
DPC: daily population contribution
PS: population size
Prev: prevalence in the given population
Vol: the average volume of urine shed per day
IQR: interquartile range
