# Supplementary material for: Meta-analysis to estimate the load of Leptospira excreted in urine: beyond rats as important sources of transmission in low-income rural communities
Source: BMC Res Notes. 2017 Jan 28;10:71. doi: 10.1186/s13104-017-2384-4 (PMC5273803; doi:10.1186/s13104-017-2384-4)
Supplement: Supplementary file 1 — Additional file 1: Figure S1. Study flow diagram. [file 13104_2017_2384_MOESM1_ESM.docx]

**Study Flow Diagram**

## Studies

## Included

## Study

## Eligibility

## Screening

**Of Records**

## Identification

**Of Records**

We searched Pubmed and Web of Science on October 24th, 2015 using the terms “Leptospira AND ((Shedding) OR (Excretion) OR (Leptospiruria))” without restrictions on publication date.
(n = 235)

Full-text articles excluded: Studies that didn’t include quantity of Leptospira in urine.
(n = )

Studies included in qualitative synthesis: only those that reported the quantity of Leptospira in urine of animals infected naturally or experimentally (n = 14)

Records excluded: papers not about leptospirosis, did not quantify Leptospira in urine, were in languages other than English or Spanish.
(n = 126)

Studies included in quantitative synthesis (meta-analysis)
(n = 14)

Full-text articles assessed for eligibility.
(n = 30)

Records screened.
(n = 156)

Records after duplicates removed.
(n = 156)
