# Supplementary material for: Meta-analysis to estimate the load of Leptospira excreted in urine: beyond rats as important sources of transmission in low-income rural communities
Source: BMC Res Notes. 2017 Jan 28;10:71. doi: 10.1186/s13104-017-2384-4 (PMC5273803; doi:10.1186/s13104-017-2384-4)
Supplement: Supplementary file 2 — Additional file 2: Table S1. Leptospira quantity data extracted from the articles analyzed in this meta-analysis. [file 13104_2017_2384_MOESM2_ESM.docx]

**Table S1.** *Leptospira* quantity data extracted from the articles analyzed in this meta-analysis.

Table S1. Continuation.

Table S1. Continuation.
