# Supplementary material for: Meta-analysis to estimate the load of Leptospira excreted in urine: beyond rats as important sources of transmission in low-income rural communities
Source: BMC Res Notes. 2017 Jan 28;10:71. doi: 10.1186/s13104-017-2384-4 (PMC5273803; doi:10.1186/s13104-017-2384-4)
Supplement: Supplementary file 3 — Additional file 3: Table S2. Urine volume excreted by animals. [file 13104_2017_2384_MOESM3_ESM.docx]

**Table S2.** Urine volume excreted by animals

| **Animal** | **volume/ kg* or day** | **Reference** |
| --- | --- | --- |
| **Cows** | 2000/7 to 9 times a day | Moir et al., 2011; Oudshoorn et al., 2008; Aland et al., 2002 |
| **dogs** | 500 ml/20kg | Williams, 2004 |
| **Deer** | 560-6800 ml per day | G.M.O. Maloiy, 1969; Dusek et al., 1989 |
| **mice** | 2-10.4 ml/100g | (Stechman, Ahmad et al. 2010 |
| **rat** | 3.3 /100g | Henry, D., 1924 |
| **human** | 1700 ml per day | Armstrong, Johnson et al. 2015 |

*Animal weight
