# Supplementary material for: Meta-analysis to estimate the load of Leptospira excreted in urine: beyond rats as important sources of transmission in low-income rural communities
Source: BMC Res Notes. 2017 Jan 28;10:71. doi: 10.1186/s13104-017-2384-4 (PMC5273803; doi:10.1186/s13104-017-2384-4)
Supplement: Supplementary file 4 — Additional file 4: Table S3. Estimated quantity of Leptospira shed by animals. [file 13104_2017_2384_MOESM4_ESM.docx]

**Table S3.** Estimated quantity of *Leptospira* shed by animals.

| **Animal type** | ***Leptospira* quantity** | ***Leptospira* quantity** |
| --- | --- | --- |
|  | **per ml urine.**  **Median (min-max)** | **per day.**  **Median (min-max)** |
| **Cattle** (n=9) | 3.7x10^4^ (3x10^2^-3.7x10^4^) | 6.3x10^8^ (5.1x10^6^- 1.3x10^9^) |
| **Deer** (n=28) | 1.7x10^5^ (3.6x10^3^-1.7x10^6^) | 6.1x10^8^ (1.3x10^7^-6.3x10^9^) |
| **Rat** (n=53) | 5.7x10^6^ (5 – 8x10^8^) | 7x10^7^(6.1x10^1^-9.8x10^9^) |
| **Human**(n=43) | 7.9x10^2^ (3.2x10^1^-8.5x10^6^) | 1.3x10^6^(5.4x10^4^-1.4x10^10^) |
| **Dog** (n=37) | 1.4x10^2^ (3.5x10^1^-1.3x10^6^) | 1.6x10^5^ (4.2x10^4^-1.5x10^9^) |
| **Mice** (n=4) | 3.1x10^3^ (4.7x10^2^-1.8x10^6^) | 1.9x10^5^(2.9x10^4^-1.1x10^8^) |
